# Legius syndrome: case report and review of literature

**DOI:** 10.1186/s13052-015-0115-9

**Published:** 2015-02-08

**Authors:** Elisa Benelli, Irene Bruno, Chiara Belcaro, Alessandro Ventura, Irene Berti

**Affiliations:** University of Trieste, Trieste, Italy; Institute for Maternal and Child Health - IRCCS “Burlo Garofolo”, Trieste, Italy

**Keywords:** Café-au-lait spots, Neurofibromatosis 1, Legius syndrome

## Abstract

A 8-month-old child was referred to our Dermatologic Unit for suspected Neurofibromatosis type 1 (NF 1), because of the appearance, since few days after birth, of numerous café-au-lait spots (seven larger than 5 mm); no other sign evocative of NF 1 was found. Her family history was remarkable for the presence of multiple café-au-lait spots in the mother, the grandfather and two aunts. The family had been already examined for NF 1, but no sign evocative of the disease was found. We then suspected Legius syndrome, a dominant disease characterized by a mild neurofibromatosis 1 phenotype. The diagnosis was confirmed by the finding of a mutation in *SPRED1* gene, a feedback regulator of RAS/MAPK signaling. Here, we discuss the differential diagnosis of cafè-au-lait spots and we briefly review the existing literature about Legius syndrome.

## Background

Cafè-au-lait macules (CALMs) are well-circumscribed, light brown macules that can develop at birth or later in life. They are common findings in Pediatrics, but they can be a sign of many different conditions: they can be isolated or related to a more complex syndrome. In the last case it’s important to look for other signs evocative of a syndrome in the patient or in his family.

We describe a healthy 8-month-old child with multiple cafè-au-lait spots. Her family history was characterized by the presence of CALMs in many members, but other manifestations typical of Neurofibromatosis type 1 (NF 1) were not described. Because of the presence of isolated CALMs in many family members, we hypothesized a Legius syndrome, an autosomal dominant disease characterized by a mild NF 1 phenotype.

## Case

**Figure 1:**
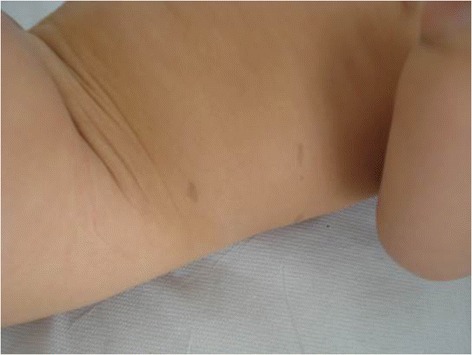
**Some of the child café-au-lait macules.**

A 8-month-old female child was referred to our Dermatologic Unit for suspected Neurofibromatosis type 1 (NF 1), because of the appearance of several café-au-lait macules (CALMs) since first days of life. Her medical history was otherwise unremarkable and growth and development were appropriate for age. At physical exam, we noticed that the child had multiple CALMs, of which 7 were larger than 5 mm (Figure 1). No other clinical feature of NF 1 was found (i.e. Lisch nodules, axillar or inguinal freckles, neurofibromas, gliomas).

**Figure 2:**
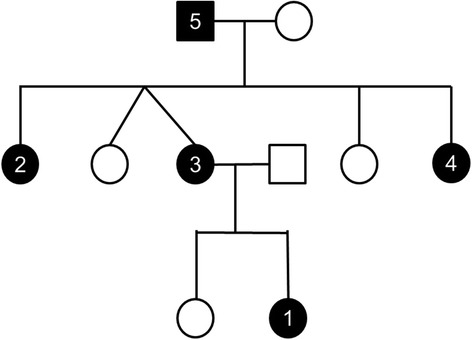
**The child’s family tree.** N.1: the patient (a 8-month-old female child) had multiple cafè-au-lait spots; n. 2 – 3 – 4–5: two aunts, the mother and the grandfather had multiple cafè-au-lait spots; n.4: the youngest aunt had also inguinal freckles and in the past had suffered of epilepsy and mild scholar learning disabilities.

**Figure 3:**
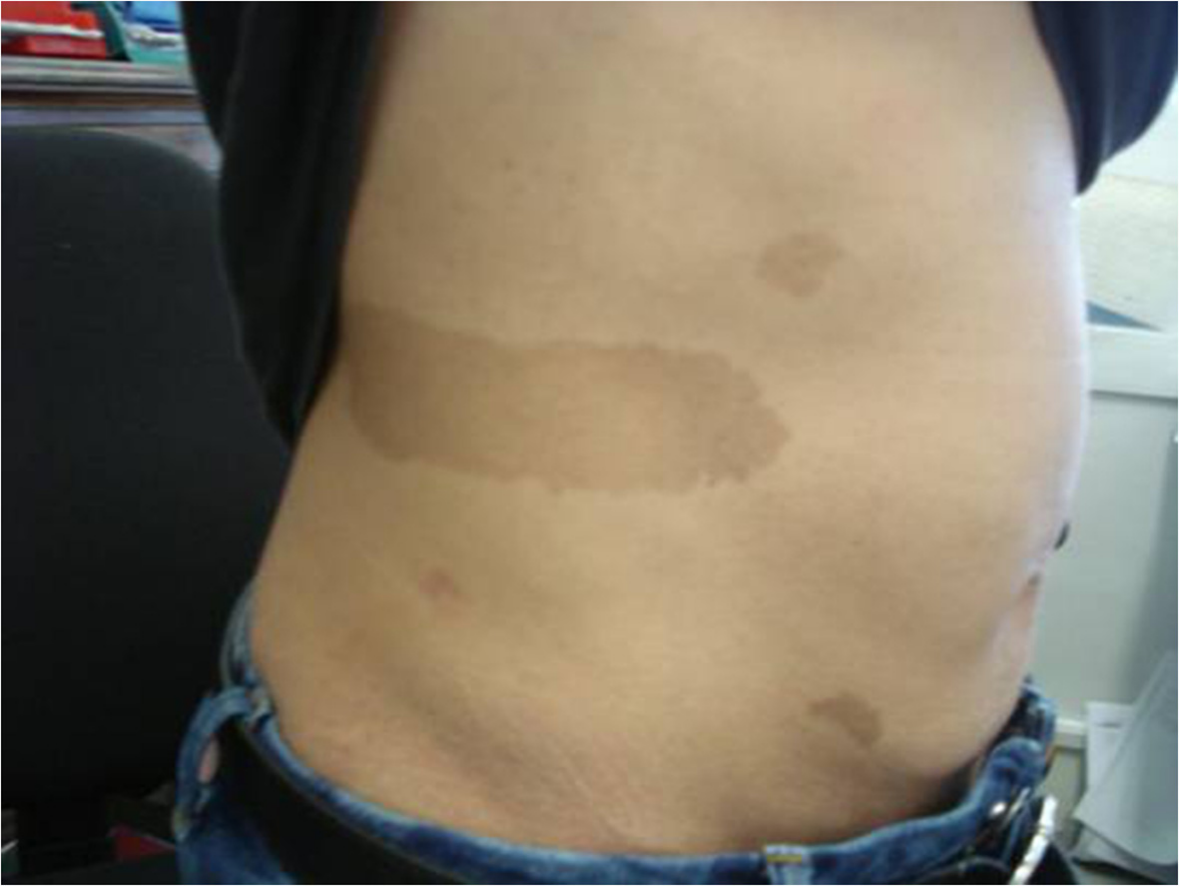
**One of the mother’s café-au-lait macules.**

On the other hand, the patient’s family history was instead interesting (Figure 2): the mother, the grandfather and two aunts had also multiple CALMs. In particular, our patient’s mother presented more than 6 café-au-lait spots larger than 15 mm (Figure 3). The youngest aunt had also inguinal freckles and during childhood she had suffered from benign epilepsy and mild scholar learning disabilities, achieving a complete healing with growth. In childhood our patient’s mother and her family were examined for NF 1, but no Lisch nodule or neurofibroma was found. During those examinations, macrocephaly was noticed in our patient’s mother. Since then, none of the family members have developed any known complications of NF 1.

CALMs are common findings in healthy new-borns and in children, but the presence of multiple macules, large segmental lesions or the association with facial dysmorphism, other cutaneous anomalies or unusual findings at the physical exam, should arise the suspicion of a more severe disease [1].

In our case, family history was highly suspicious for an autosomal dominant disease, because the clinical sign (i.e. café-au-lait spots) was present in every generation. CALMs are usually the first sign of NF 1 and it is well known that the other clinical manifestations of NF 1 became evident with age [2]. For this reason, we could not exclude NF 1 right away in our patient, but it seemed unlikely because of the lack of the other typical manifestations in the older family members (i.e. Lisch nodules, neurofibromas, gliomas). However, some patients with a NF1 phenotype characterized only by cutaneous signs and specific mutations in NF1 gene (c.2970-2972 delAAT;p.990delM) [3] (c.5425C > T;p.Arg1809Cys) [4], have been recently described. Finally, neither the child nor the other family members have nevus anemicus or juvenile xanthogranuloma, which have been recently described be more frequent in patients with NF 1 [5]. Other dominant autosomal diseases presenting with CALMs were excluded because of the lack of other associated manifestations. The McCune Albright syndrome, for example, is characterized by many endocrinopathies (in particular by precocious puberty) and polyostotic fibrous dysplasia, but none of the family members have ever presented one of these manifestations. The LEOPARD syndrome, another autosomal dominant disease, is instead mainly characterized by “lentigines” (freckles), even if CALMs can also be present. Moreover, this syndrome is characterized by electrocardiography conduction abnormalities, ocular hypertelorism, pulmonary stenosis, abnormal genitalia, retarded growth and sensorineural deafness.

Because of the lack of any sign other than CALMs and freckles, we suspected Legius syndrome (LS), a dominant disease characterized by a mild neurofibromatosis phenotype [6]. The diagnosis in our patient, her mother, the grandfather and in the youngest aunt (the other aunt was not willing for tests), was confirmed by the finding of a pathogenic mutation, resulting in a stop codon, in the *SPRED1* gene (c.973C > T; p.Arg325*), a feedback regulator of RAS/MAPK signaling and the responsible of the disease [7].

Multiple café-au-lait spots, with or without freckles and macrocephaly, characterize LS, whereas other complications of NF 1 are absent [8]. As reported in a recent review, other malformations are occasionally found in patients with LS, such as Noonan-like face, *pectus excavatum* or *carinatum* and lipomas [9]. Learning disabilities and behavioral problems are also possible manifestations of the disease, as found by Denayer and al., who studied 15 patients with LS [10]**.** The same authors reported that cognitive disabilities in LS are less severe than in NF 1.

The disease was first described in 2007 by Brems and colleagues, who identified that a heterozygous mutation in *SPRED1* gene was responsible of this mild neurofibromatosis phenotype [11]. After 2007, more than 200 cases have been reported [6] and it has been demonstrated that up to 2% of patients fulfilling diagnostic criteria for NF 1, without NF1 gene mutations, have instead *SPRED1* mutations [7]. *SPRED1* acts as a negative regulator of RAS pathway and interacts with neurofibromin, the product of the NF1 gene [12]. Up to date, many different allelic variants have been found, but no genotype-phenotype correlation has been noted.

## Conclusion

Pediatricians must be aware of LS in all children with CALMs, particularly in those without a family history for NF1 complications. Identifying this condition is important, to avoid the stress related to a NF1 diagnosis, but also to start early cognitive and behavioral problems screening.

Written informed consent was obtained from the patient’s parent for publication of this Case report and any accompanying images. A copy of the written consent is available for review by the Editor-in-Chief of this journal.

## Abbreviations

CALMs: Café-au-lait macules
NF 1: Neurofibromatosis type
LS: Legius syndrome
